# Molecular and Metabolic Insights into Anthocyanin Biosynthesis for Leaf Color Change in Chokecherry (*Padus virginiana*)

**DOI:** 10.3390/ijms221910697

**Published:** 2021-10-02

**Authors:** Xiang Li, Yan Li, Minghui Zhao, Yanbo Hu, Fanjuan Meng, Xingshun Song, Mulualem Tigabu, Vincent L. Chiang, Ronald Sederoff, Wenjun Ma, Xiyang Zhao

**Affiliations:** 1 State Key Laboratory of Tree Genetics and Breeding, School of Forestry, Northeast Forestry University, Harbin 150040, China; lx2019@nefu.edu.cn (X.L.); ly2019@nefu.edu.cn (Y.L.); zhaomh@nefu.edu.cn (M.Z.); charminghu@nefu.edu.cn (Y.H.); mengfan2005@nefu.edu.cn (F.M.); xssong@nefu.edu.cn (X.S.); vchiang@ncsu.edu (V.L.C.); 2 Southern Swedish Forest Research Centre, Swedish University of Agricultural Sciences, 230 53 Alnarp, Sweden; mulualem.tigabu@slu.se; 3 Forest Biotechnology Group, Department of Forestry and Environmental Resources, North Carolina State University, Raleigh, NC 27695, USA; ron_sederoff@ncsu.edu; 4 State Key Laboratory of Tree Genetics and Breeding, Key Laboratory of Tree Breeding and Cultivation of State Forestry Administration, Research Institute of Forestry, Chinese Academy of Forestry, Beijing 100091, China; 5 College of Forestry and Grassland, Jilin Agricultural University, Changchun 130118, China

**Keywords:** *Padus virginiana*, leaf color, anthocyanin biosynthesis, transcriptomics, metabolomic

## Abstract

Chokecherry (*Padus virginiana* L.) is an important landscaping tree with high ornamental value because of its colorful purplish-red leaves (PRL). The quantifications of anthocyanins and the mechanisms of leaf color change in this species remain unknown. The potential biosynthetic and regulatory mechanisms and the accumulation patterns of anthocyanins in *P. virginiana* that determine three leaf colors were investigated by combined analysis of the transcriptome and the metabolome. The difference of chlorophyll, carotenoid and anthocyanin content correlated with the formation of *P. virginiana* leaf color. Using enrichment and correlation network analysis, we found that anthocyanin accumulation differed in different colored leaves and that the accumulation of malvidin 3-O-glucoside (violet) and pelargonidin 3-O-glucoside (orange-red) significantly correlated with the leaf color change from green to purple-red. The flavonoid biosynthesis genes (*PAL*, *CHS* and *CHI*) and their transcriptional regulators (*MYB*, *HD-Zip* and *bHLH*) exhibited specific increased expression during the purple-red periods. Two genes encoding enzymes in the anthocyanin biosynthetic pathway, UDP glucose-flavonoid 3-O-glucosyl-transferase (*UFGT*) and anthocyanidin 3-O-glucosyltransferase (*BZ1*), seem to be critical for suppressing the formation of the aforesaid anthocyanins. In PRL, the expression of the genes encoding for UGFT and BZ1 enzymes was substantially higher than in leaves of other colors and may be related with the purple-red color change. These results may facilitate genetic modification or selection for further improvement in ornamental qualities of *P. virginiana*.

## 1. Introduction

*Padus virginiana* L. ‘Canada Red’, is an ornamental tree species from the Rosaceae family [1]. *P. virginiana* originated in Northeast North American and has been widely introduced in Shanxi Province, Hebei Province and Northeast China. Due to its stress resistance, adaptability and ornamental value, *P. virginiana* is widely used as a colored leaf tree in greenbelt landscape. The leaf color of *P. virginiana* is rich in different growth seasons and varies from green and purple to purplish-red. The flowers, fruits, bark and leaves of *P. virginiana* are used as medicines, and the fruit contains proteins, vitamins, sugars, anthocyanins and flavonoids which act as antioxidants and can also relieve thirst and clear intestines [2]. *P. virginiana* has strong cold, drought and heat resistance. It can survive in extremely low temperature (−40 °C) and extremely high temperature (40 °C) under natural growth conditions. It is a suitable colored-leaf tree for urban landscaping in tropical and cold temperate region [3]. Previous research has focused on nursery propagation, photosynthetic physiology and stress resistance in *P. virginiana* [4,5]. However, no reports were related to the dynamic changes, transcriptional control and metabolic pathways of anthocyanins in leaves of *P. virginiana*.

The polymorphic colors of plants are determined by a combination of several classes of pigments. The major pigments in plants are the chlorophylls (green), carotenoid (from yellow to red) and anthocyanins (from red to purple), which provide the foundation for coordinated regulation of plant tissue color [6]. Anthocyanins play a key role in leaf and fruit coloration. The biosynthetic pathway of anthocyanin has been well elucidated in some plants, such as in *Arabidopsis thaliana* [7]. Anthocyanins are flavonoid with special molecular structures and strong water solubility. Anthocyanidins are grouped in six categories according to differences of molecular structure: malvidin, petunidin, pelargonidin, peonidin, delphinidin and cyanidin. Anthocyanins are significant secondary metabolites and widely distributed in leaves, flowers, fruits and seeds of many plants, for example, in *Fragaria ananassa* Duch. [8], *Vitis vinifera* L. [9] and *Morus alba* L. [10]. Anthocyanins in plants maintain various colors, protect photosynthesis, resist oxidation and moderate stress. In addition, anthocyanins can delay senescence by scavenging free radicals and inhibiting tumorigenesis.

The anthocyanin biosynthetic pathway is a branch of phenylpropanoid biosynthesis that shares its initial steps with the pathways for many metabolites, such as the monolignols hydroxycinnamic acids, sinapoyl esters, coumarins and stilbenes [11]. Phenylpropanoid biosynthesis involves many structural genes and gene families encoding enzymes such as phenylalanine ammonialyase (PAL), the initial enzyme for all phenylpropanoid biosynthesis by deamination of phenylalanine to yield cinnamic acid. Cinnamic acid 4-hydroxylase (C4H) adds a hydroxyl group to produce p-coumaric acid, which is, in turn, activated by 4-coumarate CoA ligase (4CL). The key branch point from monolignol biosynthesis to the anthocyanins is catalyzed by chalcone synthase (CHS), which condenses two molecules of p-coumaric acid through their propanoid side chains to produce naringenin chalcone, a yellow flower pigment and the precursor for all anthocyanins. In addition, the genes involved in anthocyanin biosynthesis are also regulated by MBW, a plant transcription factor complex composed of MYB–bHLH–WD40 proteins [12]. Understanding the biosynthesis of anthocyanins in model and non-model plant species should better account for tissue color variation.

In recent years, the application of “multi-omics” and high-throughput genome sequence techniques accelerated the studies of functional traits in plants and the identification of specific metabolic pathways in plants, thereby enabling the characterization and mining of key genes in phenylpropanoid biosynthesis [13]. Transcriptome analysis followed by the development of high-throughput sequencing is now a widely applicable technology for studying plant genetic regulation. It is the vital link between genome genetic information and the proteome. However, it cannot detect the changes of plant metabolism and the mechanisms of key genes regulating various metabolic pathways. Metabolomics, the comprehensive identification and quantification of populations of metabolites, is able to describe the dynamic changes of endogenous metabolites before and after differentiation in development, gene mutation or environmental stress. The core strategy is to quantify small molecules such as nucleosides, terpenoids, alkaloids, amino acids and sugars and to analyze the changes in differential abundance of metabolites in different growth stages and growth conditions. The changes in metabolite concentrations provide important genetic information for the functional description of complex metabolic networks and their modification by advanced breeding. Combined transcriptomics and metabolomics can not only result in greater understanding of the expression of specific genes but also result in the identification of functional genes related to biological characteristics from the changes in the level of metabolites and to reveal the regulatory mechanisms of specific genes. The integrated analysis of transcriptomes and metabolomes can now be employed to identify genes that determine leaf color, fruit development and pigment deposition [14,15]. Today, the functions of key genes that directly determine and coordinate anthocyanin biosynthesis during leaf color change in *P. virginiana* remain unclear.

In this study, transcriptomic and metabolomic technologies were used to identify differentially expressed genes (DEGs) and to reveal the changes in anthocyanin biosynthesis that determine leaf color changes in *P. virginiana*. Through RNA-seq abundance and high-performance liquid chromatography (HPLC), we investigated the transcript profiles of the green, purple and purplish-red leaves in order to elucidate the relationship of structural genes encoding the enzymes for anthocyanin biosynthesis and color formation in leaves of *P. virginiana*. Our results provide new insights for the identification of the functional genes and metabolites with leaf color change, which lays a foundation for color improvement by advanced breeding technology in *P. virginiana* and other colored-leaf plants.

## 2. Results

### 2.1. Pigment Content Analysis

**Figure 1:**
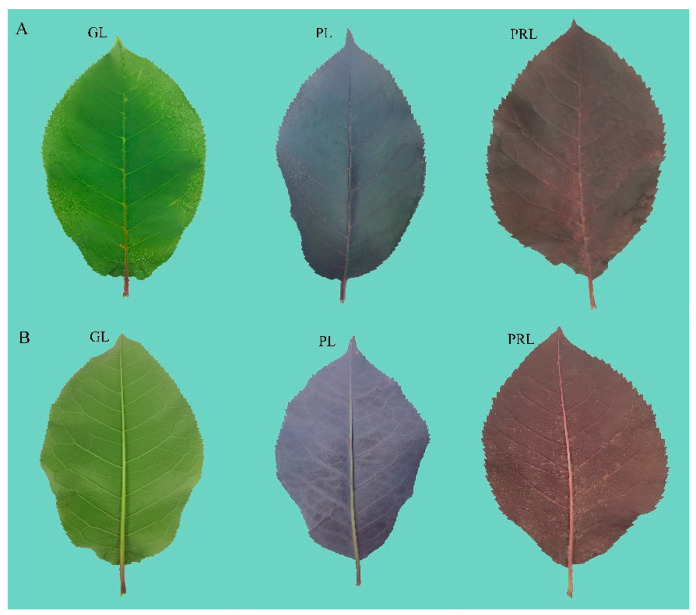
Phenotypes of *P. virginiana* green (GL), purple (RL) and purple-red (PRL) leaf sides on the adaxial (**A**) and abaxial (**B**).

To observe the phenotype and physiology during the color change of *P. virginiana*, the green leaves (GL), purple leaves (PL) and purplish-red leaves (PRL) were collected at the same developmental stage. The color differences are obvious between the “green”, “purple” and “purplish-red” leaves (Figure 1A,B). Chlorophyll a and chlorophyll b contents in green leaves were significantly higher than that in the purplish-red leaf, indicating an apparent difference in the content of leaf chlorophyll (Figure S1A–C). The carotenoid contents in green and purple leaves were higher than that in purplish-red leaf (*p* < 0.05) (Figure S1D). The difference of pigment content appears to make the *P. virginiana* leaves show different colors.

### 2.2. Identification and Quantification of Anthocyanin Components in P. virginiana

**Figure 2:**
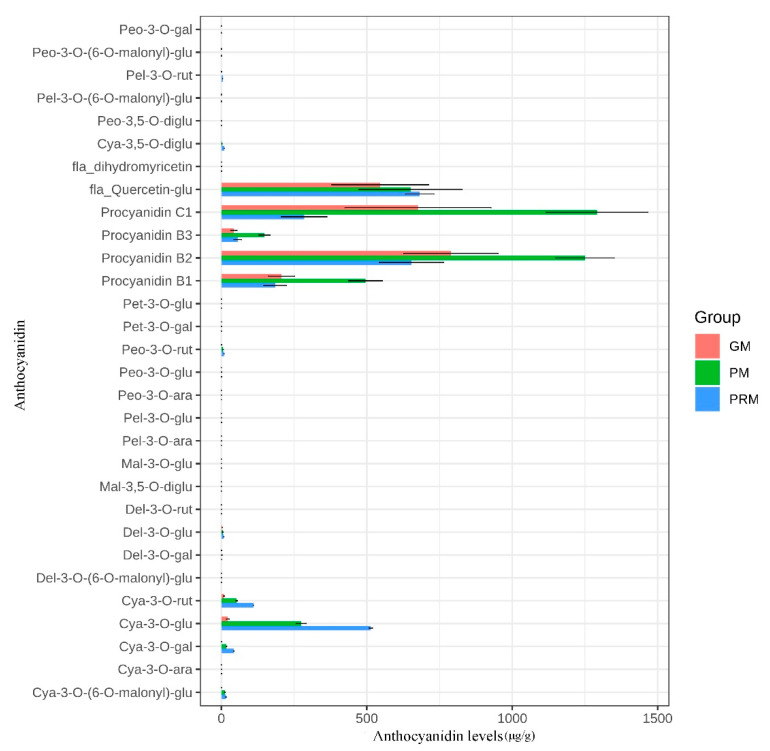
The content of metabolites detected in this study among different samples. The *x*-axis represents the anthocyanin levels (μg/g). The *y*-axis represents the anthocyanin composition obtained by high-performance liquid chromatography (HPLC). Error bars show standard error (SE) of the mean. GL, green leaves; PL, purple leaves; PRL, purple-red leaves.

**Figure 3:**
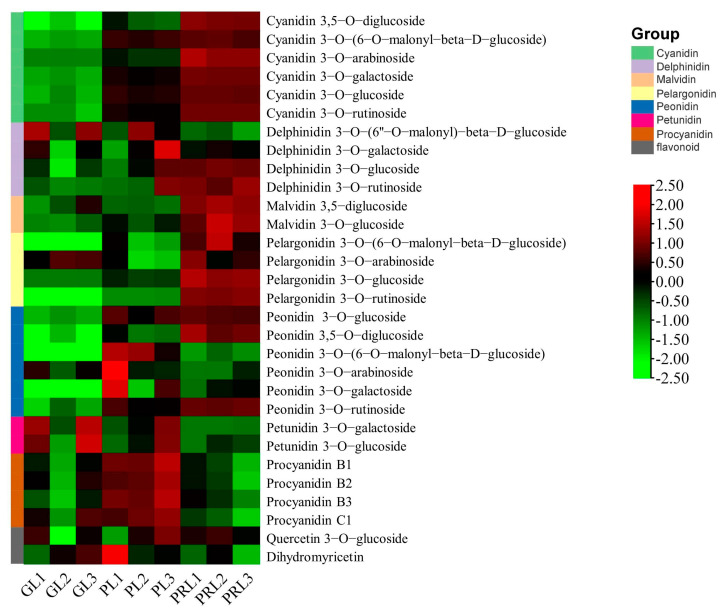
Heatmap of metabolites related to cyanidin, peonidin, delphinidin, pelargonidin, procyanidin, flavonoid, malvidin and petunidin in GL, PL and PRL. The marker on the right side of heatmap represents the names of each anthocyanin composition obtained by high–performance liquid chromatography (HPLC). Color scale from green to red in the heatmap represents the normalized metabolite contents (from low to high) using Row Z-score. GL, green leaves; PL, purple leaves; PRL, purple—red leaves.

The color of *P. virginiana* leaves at the early stage (before June) was green, whereas the leaf color at the ripening stage (after June) was purplish-red, which is a dramatic color difference [16]. At the later growth stage, our leaf samples changed from green (GL), to purple (RL) and then to purple-red (PRL). Leaves of different colors were collected and analyzed by HPLC to detect anthocyanins during leaf color transition (Figure 1A,B). The metabolites of the samples were identified and quantified by using HPLC using broadly selective metabolomic methods. A total of 30 anthocyanin related compounds were detected that may be responsible for the leaf color (Figure 2 and Figure S2). We organized them into eight groups, which are as follows: cyanidins (6), peonidins (6), delphinidins (4), pelargonidins (4), procyanidins (4), flavonoids (2), malvidins (2) and petunidins (2) (Figure 3). Cyanidin and peonidin were abundant in all leaf samples of *P. virginiana*, and procyanidin B2, procyanidin C1 and quercetin 3-O-glucoside were highest in green (GL), purple (RL) and purple-red (PRL) samples, respectively. To visualize the relationships of the metabolites from nine samples, heatmap clustering was performed by using R software (version 4.0.3) (Figure S3). The biological replicates grouped together (cluster of the column), indicating that the comparisons of phenotypes and metabolites were consistent and reliable. All of the nine samples could be grouped into two obvious classes: (1) green-colored samples (GL) and (2) deep-colored samples (PL and PRL).

### 2.3. Differentially Accumulated Anthocyanin Components of Leaf Color Change

**Figure 4:**
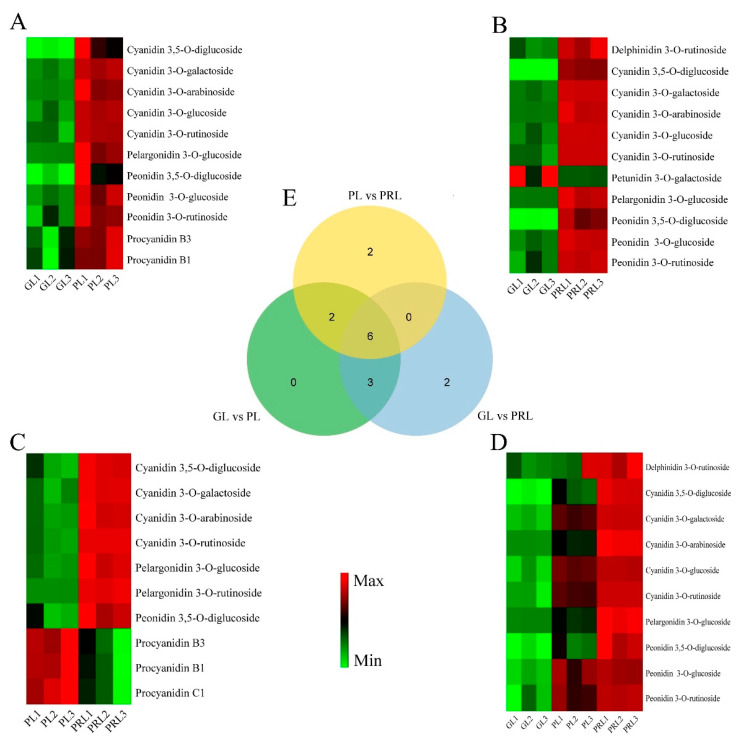
Heatmaps of differentially accumulated metabolites (DAMs) in GL vs. PL (**A**), GL vs. PRL (**B**), PL vs. PRL (**C**) and GL vs. PL–PRL. (**D**,**E**) Venn diagram of DAMs in *P. virginiana*. GL, green leaves; PL, purple leaves; PRL, purples-red leaves. The color scale from Min (green) to Max (red) refer to the metabolite contents from low to high. Identification of differentially accumulated metabolites (DAMs) between four comparison groups was performed by variable importance in projection (VIP) values (VIP ≥ 1) and fold change ≥ 2 or ≤ 0.5. Venn diagram representing overlap between DAMs identified in *P. virginiana* at GL vs. PL, GL vs. PRL and PL vs. PRL.

In order to comprehensively screen the differentially accumulated metabolite (DAM) between pairs of leaf samples (GL vs. PL, GL vs. PRL, PL vs. PRL and GL vs. PL–PRL) in *P. virginiana*, the metabolites with VIP value ≥ 1 and fold change ≥ 2 or ≤ 0.5 were selected. For the GL vs. PL–PRL test, the deep colored samples were pooled. The differences in metabolite composition and their expression levels, shown in a Venn diagram and heat map, indicate that the metabolites were significantly different between the four comparisons, including 11 (11 upregulated), 11 (10 upregulated and 1 downregulated), 10 (seven upregulated and three downregulated) and 10 (10 upregulated) anthocyanins in GL vs. PL, GL vs. PRL, PL vs. PRL and GL vs. PL–PRL, respectively (Figure 4, Table S1). Among the DAMs identified in the four groups, the anthocyanin metabolites mainly consist of (1) cyanidin, pelargonidin, delphinidin, procyanidin, peonidin and petunidin. The most abundant anthocyanins in the green-colored and purple-colored samples were cyanidin and peonidin, especially the cyanidin 3-O-arabinoside, cyanidin 3-O-galactoside, cyanidin 3-O-rutinoside, peonidin 3-O-glucoside and peonidin 3-O-rutinoside, which play a significant role in the leaf color change in *P. virginiana*. The Venn diagram showed that six anthocyanins were common in the comparison of three sample types, and they were significantly enriched in anthocyanin biosynthesis (ko00942). The anthocyanins identified in the four comparison groups showed similar accumulation (Table S2). We propose that the DAMs found in anthocyanin biosynthesis may contribute to the leaf color change in *P. virginiana*.

### 2.4. Statistical Analysis of Transcriptome Data

Nine cDNA libraries constructed from total RNA from the green (GL), purple (PL) and purple-red (PRL) leaves were sequenced on the Illumina HiSeq 2000 platform to obtain variation information on transcript identity and abundance during leaf color change. A total of 450,429,372 raw reads and 437,023,138 clean reads from nine samples were obtained. Of the clean reads, the average fragments scoring Q20 and Q30 were 98.2% and 94.5%, respectively. The GC content ranged from 0.456 to 0.460, with an average of 0.458. A total of 74.5% of the clean reads were mapped uniquely against the assembled genome (Table S3). BLAST alignment was performed on unigene sequences and common databases for functional annotations, including SwissProt, KEGG, NR, GO, TREMBL, KOG and Pfam database. A number of 239,816 unigenes were successfully annotated, in which 187,299 unigenes (78.1%) were annotated in at least one database (Table S4). Based on the fragments per kilobase of exon per million fragments mapped (FPKM) values, principal components analysis (PCA) showed that the biological replicates of PRL samples clustered together and were significantly separated from the two other samples (GL and PL) (Figure S4). Based on the combined transcriptome and metabolome data, we suggested that there are different gene expressions and metabolite abundance patterns between the green/purple and purple-red leaf samples in response to leaf color change in *P. virginiana*.

### 2.5. Differentially Expressed Genes in Leaves of P. virginiana

**Figure 5:**
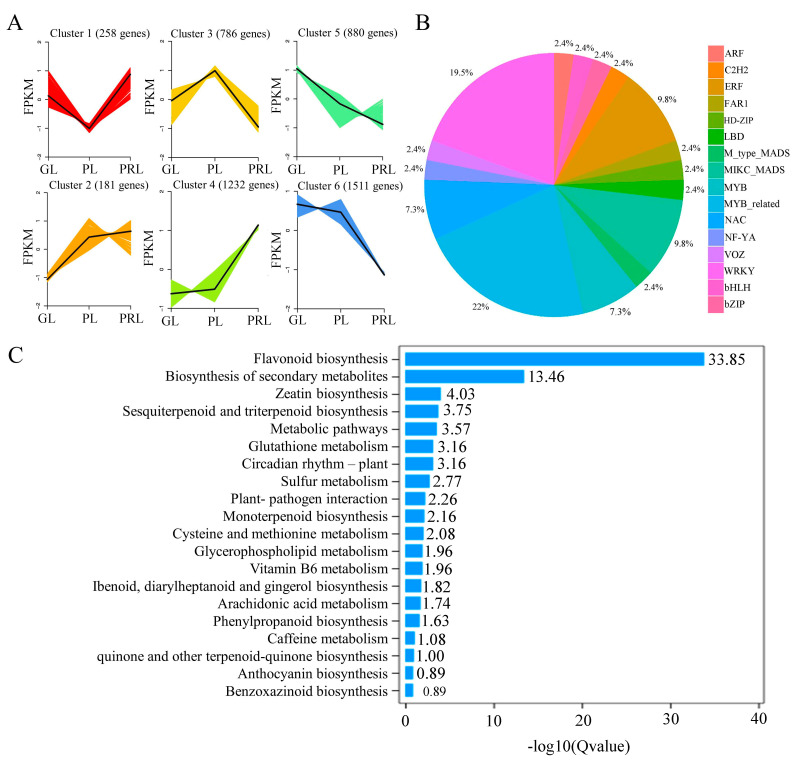
Gene regulation during leaf color change. (**A**) K-means cluster analysis of co-expression genes and their expression patterns. (**B**) The DEGs involved in transcriptome factor enriched in cluster 4. (**C**) KEGG enrichment bar plot of DEGs in cluster 4. Cluster 4 represents the expression pattern of 1232 co-expression genes identified in the K-means cluster analysis. FPKM represents the fragments per kilobase per million.

To infer the underlying transcriptional regulatory relationship of DEGs and identify co-expressed genes, K-means cluster analysis was performed using the FPKM values of GL, PL and PRL transcriptome data. Six clusters of DEGs were found during leaf color change, suggesting that each cluster had a similar molecular function (Figure 5A). Cluster one consists of 258 genes. The expression first decreases and then increases from green leaf to purple-red leaf. Cluster three contains 786 genes with a pattern opposite to cluster one, suggesting an activation/inhibition relationship. A number of 181 genes were identified in cluster two, displaying a more modest increase from green leaf to purple-red leaf. A significant increase in expression is observed for cluster 4 (1232 genes). Enrichment analysis of the genes in cluster four indicates that they play a role in flavonoid and phenylpropanoid biosynthesis during leaf color change in *P. virginiana* related to some common transcription factors (TFs), such as MYB, WRKY, NAC, MADS-box and ERF (Figure 5B,C). A total of 880 genes in cluster five exhibited a negative correlation with leaf color change. In total, 1511 genes in cluster six showed an abrupt decline in their expression, especially for the purple to purple-red transition during leaf development.

DESeq2 software was used to identify DEGs. A total of 6710 DEGs (2236 upregulated and 4474 downregulated) were found in the three comparison groups of leaf samples (Table S5; Figure S5). There are 199 DEGs (97 upregulated and 102 downregulated) in GL vs. PL, 3336 DEGs (1079 upregulated and 2257 downregulated) in GL vs. PRL and 3175 DEGs (1060 upregulated and 2115 downregulated) in PL vs. PRL. Only 11 DEGs were common to all three comparison groups, suggesting that these conserved genes may be associated with leaf color change. The GO and KEGG functional annotation and pathway analyses found that the DEGs in the GL vs. PRL group were enriched in KEGG terms ko01100 (for metabolic pathways), ko01110 (for biosynthesis of secondary metabolites), ko00940 (for phenylpropanoid biosynthesis) and ko00941 (flavonoid biosynthesis). These pathways also occupied the top enriched KEGG pathway in PL vs. PRL, suggesting that these DEGs may play a significant role in leaf color-related biosynthesis, especially for phenylpropanoids and flavonoids. Only ko00073 (cutin, suberin and wax biosynthesis) and ko00941 (flavonoid biosynthesis) pathways were significantly changed in GL vs. PL (Table S6).

### 2.6. Phenylpropanoid, Flavonoid and Anthocyanidin Biosynthesis Pathway during Leaf Color Change

**Figure 6:**
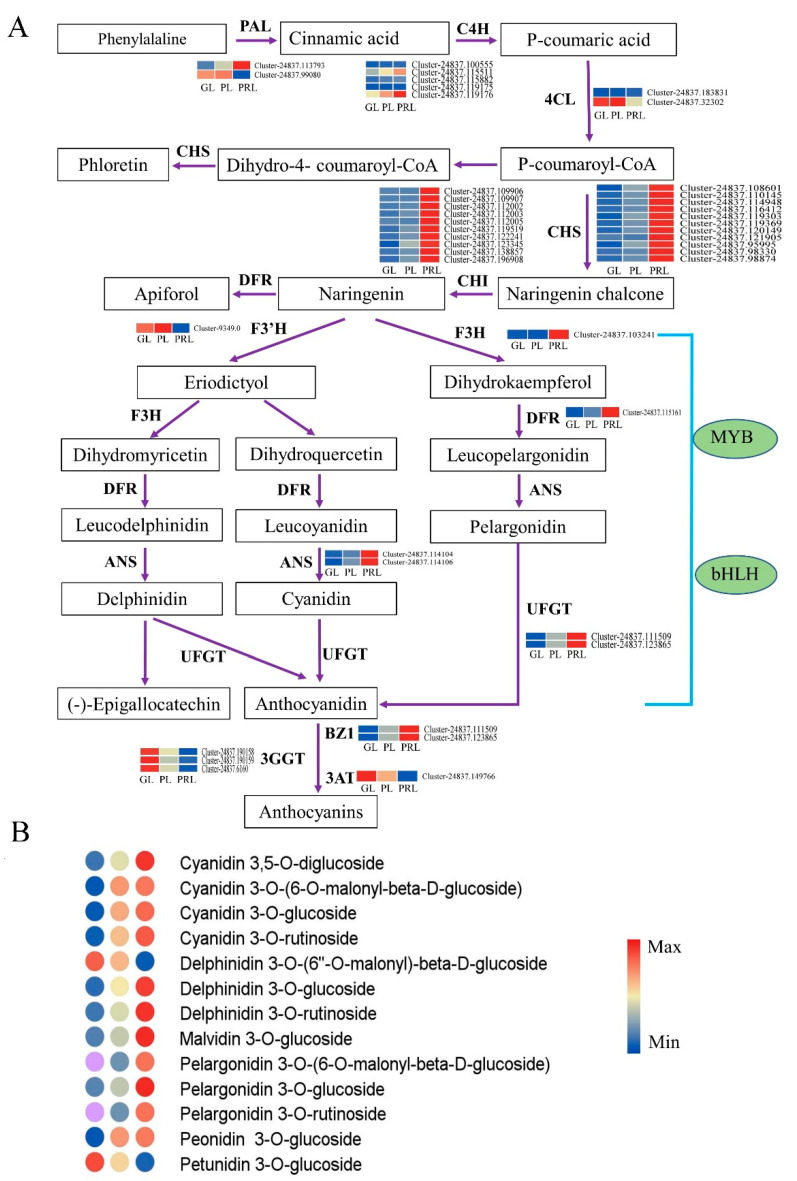
The expression of genes in the phenylpropanoid and flavonoid biosynthetic pathways in *P. virginiana* leaf. (**A**) Reconstruction of the anthocyanins biosynthetic pathway with the differentially expressed structural genes and their regulators. The differentially expressed genes (DEGs) were identified by an adjusted *p*-value < 0.05 and |log_2_ fold change (FC)| ≥ 1. (**B**) The differentially accumulated metabolites (DAMs) in the anthocyanin biosynthetic pathway. The DAMs were identified by projection (VIP) values (VIP ≥ 1) and fold change ≥ 2 or ≤ 0.5. The color scale from Min (blue) to Max (red) refer to the metabolite contents from low to high. The cluster marker on the right side of heatmap represents the names of each gene. GL, green leaf; PL, purple leaf; PRL, purple-red leaf; PAL, phenylalanine ammonia-lyase; C4H, cinnamic acid 4-hydroxylase; 4CL, 4-coumarate CoA ligase; CHS, chalcone synthase; CHI, chalcone isomerase; DFR, dihydroflavonol 4-reductase; F3H, lavanone 3-hydroxylase; F3′H, lavonoid 3′-hydroxylase; ANS, anthocyanidin synthase; UFGT, UDP glucose-flavonoid 3-O-glcosyl-transferase; BZ1, anthocyanidin 3-O-glucosyltransferase; 3GGT, 3-O-glucoside 2″-O-glucosyltransferase; 3AT, 3-O-glucoside-6″-O-malonyltransferase; MYB, v-myb avian myeloblastosis viral oncogene homolog; bHLH, basic helix-loop-helix.

Leaf color in plants is determined by the pigments in the vacuole, especially the anthocyanins. Enrichment analysis revealed that there are 74 DEGs encoding 10 enzymes in the phenylpropanoid, flavonoid and anthocyanidin biosynthetic pathways that are differentially expressed (Figure 6A). The transcript abundance of six key structural gene families including *CHS* (11 DEGs), *CHI* (10 DEGs), *F3H* (1 DEG), *DFR* (1 DEG), *ANS* (2 DEGs) and *UFGT* (2 DEGs) were higher in PRL than in GL and PL, consistent with the high-abundance of anthocyanins and leaf color change (Figure 6A). The expression of one *F3′H* gene (*Cluster-9349.0*) was significantly downregulated from naringenin to eriodictyol in PRL compared with GL and PL. In the biosynthetic steps from cinnamic acid to p-coumaric acid and p-coumaroyl-CoA, the expression of three *C4H* (*Cluster-24837.100555, Cluster-24837.115882* and *Cluster-24837.119175*) genes and one *4CL* gene (*Cluster-24837.183831*) had no significant change. For *PAL* genes, one gene (*Cluster-24837.113793*) was activated in PRL but another one (*Cluster-24837.99080*) was activated in PL and PRL stages. Most of the MYB and bHLH TFs were upregulated either in GL vs. PRL or in PL vs. PRL (Table S7). In contrast, the majority of the AP2/ERF TFs were downregulated. Three genes including *BZ1* (*Cluster-24837.111509* and *Cluster-24837.123865*), *3GGT* (*Cluster-24837.190158*, *Cluster-24837.190159* and *Cluster-24837.6160*) and *3AT* (*Cluster-24837.149766*) were found in anthocyanidin biosynthesis, and all *BZ1* genes were significantly upregulated in purple-red leaf. The differentially accumulated metabolites (DAMs) in the anthocyanin biosynthetic pathway (ko00941) include four cyanidins (upregulated), three pelargonidins (upregulated), three delphinidins (two upregulated and one downregulated), one malvidin (upregulated), one petunidin (downregulated) and one peonidin (upregulated) (Figure 6B). The genes identified in flavonoid and anthocyanidin biosynthesis play a crucial role, and the MYB, bHLH and AP2/ERF TFs coordinately regulate the structural genes, which promotes the formation of anthocyanins (cyanidin 3,5-O-diglucoside; cyanidin 3-O-(6-O-malonyl-beta-D-glucoside); cyanidin 3-O-glucoside; cyanidin 3-O-rutinoside; delphinidin 3-O-glucoside; delphinidin 3-O-rutinoside; malvidin 3-O-glucoside; pelargonidin 3-O-(6-O-malonyl-beta-D-glucoside); pelargonidin 3-O-glucoside; pelargonidin 3-O-rutinoside; and peonidin 3-O-glucoside, resulting in the leaf color change from green to purple-red.

### 2.7. Chlorophyll Biosynthesis Pathway during Leaf Color Change

**Figure 7:**
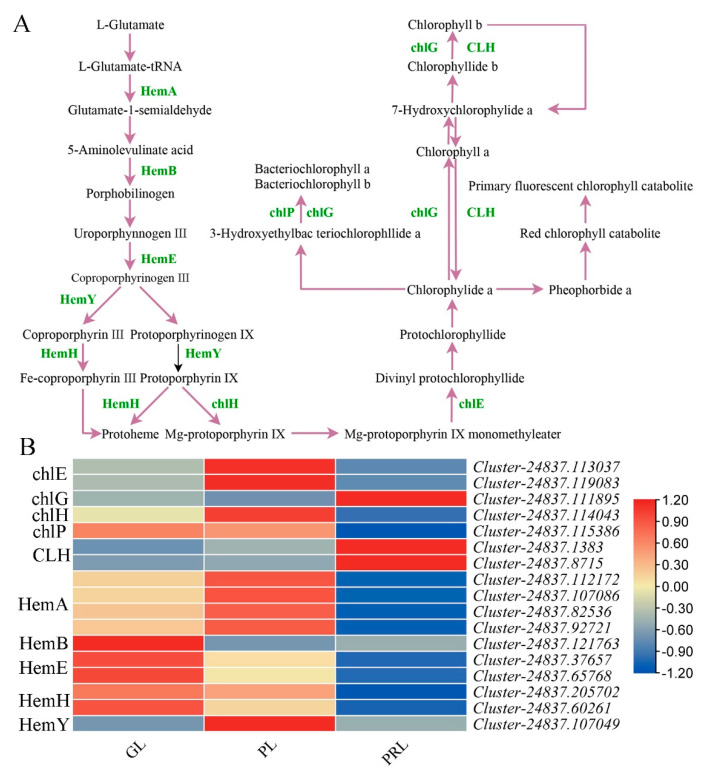
Biosynthetic pathway of chlorophyll in *P. virginiana* leaf color change. (**A**) Reconstruction of the chlorophyll biosynthetic pathway with the differentially expressed structural genes (**B**). Heatmap of the differentially expressed genes (DEGs) involved in chlorophyll biosynthetic pathway. Gene expression was scaled using Z-scores of fragments per kilobase of exon per million fragments mapped (FPKM) for mean valued of three biological replicates in heatmaps. The cluster marker on the right side of heatmap represents the names of each gene. GL, green leaf; PL, purple leaf; PRL, purple-red leaf; HemA, glutamyl-tRNA reductase; HemB, prophobilinogen synthase; HemE, uroporphyrinogen decarboxylase; HemY, copropophyrinogen III oxidase; HemH, ferrochelatase; chlH, magnesium chelatase subunit H; chlE, anaerobic magnesium-protoporphyrin IX monomethyl ester cyclase; chlG, chlorophyll a synthase; chlP, geranylgeranyl reductase; CLH, chporophyllase.

The key genes related to chlorophyll biosynthesis were identified. There are 17 DEGs encoding 10 enzymes, including *ch1* (two DEGs), *chl-G* (one DEG), *ch1-H* (one DEG), *ch1-P* (one DEG), *CL-H* (two DEGs), *HemA* (four DEGs), *HemB* (one DEG), *HemE* (two DEGs), *HemH* (two DEGs) and *HemY* (one DEG), in chlorophyll biosynthesis during leaf color change (Figure 7A). Chlorophyll biosynthesis begins with L-glutamate-tRNA and the *HemA* gene product (glutamyl-tRNA reductase) that converts L-glutamate-tRNA to glutamate-1-semialdehyde. The *HemA* gene was significantly upregulated in GL and PL (Figure 7B). With the exception of *ch1-G* and *CL-H*, the remaining genes involved in chlorophyll biosynthesis were highly expressed in the GL and PL samples compared with PRL. These crucial genes have similar expressions and may play a role in chlorophyll formation and leaf color change in *P. virginiana*.

### 2.8. Analysis of Transcriptome Factors

In addition to the structural genes discussed above, anthocyanidin biosynthesis is also regulated by transcription factors (TFs). Table S8 lists the potential anthocyanidin biosynthesis TFs in *P. virginiana*. In total, 15 DEGs (six upregulated and nine downregulated), 157 DEGs (67 upregulated and 90 downregulated) and 167 DEGs (62 upregulated and 105 downregulated) differentially expressed TFs were identified in GL vs. PL, GL vs. PRL and PL vs. PRL by comparing the PlnTFDB and PlantTFDB databases, respectively. The most abundant TFs were annotated as MYB, WRKY, bHLH, HD-ZIP, NAC, AP2/ERF, TCP and MADS-box factors (Table S8). Other transcription factors were also found in this study (Table S9). The largest number of differentially expressed TF (DETFs) in GL vs. PRL (18 upregulated and 12 downregulated) and PL vs. PRL (17 upregulated and 10 downregulated) was MYB. Most MYB-TFs (such as MYB3, MYB4 and MYB113) were upregulated and coordinated with the majority of structural genes of flavonoid biosynthesis during leaf color change, suggesting a positive regulatory role in leaf color change. Almost all of the bHLH-TFs (such as bHLH35, bHLH14, bHLH67 and bHLH70) were downregulated in GL vs. PRL (one upregulated and nine downregulated) and in PL vs. PRL (13 downregulated) from the green and purple to purple-red leaf, indicating a negative role in anthocyanin biosynthesis. One *bHLH* gene (*Cluster-24837.158504*) was upregulated in GL vs. PRL. Two *HD-ZIP* genes (*Cluster-24837.117097* and *Cluster-24837.25187*) were identified to have high expression in purple-red leaves compared with green leaves.

### 2.9. Correlation between DEGs and Anthocyanins

**Figure 8:**
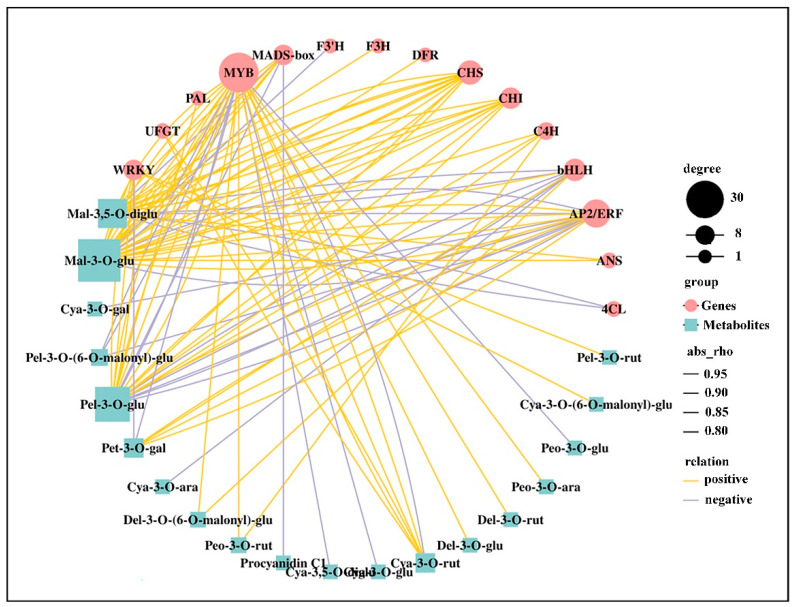
Correlation network of metabolites and genes (15 key genes) involved in phenylpropanoid and flavonoid biosynthesis in GL vs. PRL. Abs_rho represents the Pearson correlation coefficient (r). Degree represents the gene number. Relation represents the correlations with a coefficient (r) value > 0.8 (positive) or < −0.8 (negative). PAL, phenylalanine ammonia-lyase; C4H, cinnamic acid 4-hydroxylase; 4CL, 4-coumarate CoA ligase; CHS, chalcone synthase; CHI, chalcone isomerase; DFR, dihydroflavonol 4-reductase; F3H, lavanone 3-hydroxylase; F3′H, lavonoid 3′-hydroxylase; ANS, anthocyanidin synthase; UFGT, UDP glucose-flavonoid 3-O-glcosyl-transferase; MYB, v-myb avian myeloblastosis viral oncogene homolog; bHLH, basic helix-loop-helix; WRKY, “WRKY” domain genes; AP2/ERF, the APETALA2/Ethylene-responsive factor; Mal-3,5-O-diglu, malvidin 3,5-diglucoside; Mal-3-O-glu, malvidin 3-O-glucoside; Cya-3-O-gal, cyanidin 3-O-galactoside; Pet-3-O-(6-O-malonyl)-glu, petunidin 3-O-(6-O-malonyl-beta-D-glucoside); Pel-3-O-glu, pelargonidin 3-O-glucoside; Pet-3-O-gal, petunidin 3-O-galactoside; Cya-3-O-ara, cyanidin 3-O-arabinoside; del-3-O-(6-O-malonyl)-glu, delphinidin 3-O-(6′′-O-malonyl)-beta-D-glucoside; Peo-3-O-rut, peonidin 3-O-rutinoside; Cya-3,5-O-diglu, cyanidin 3,5-O-diglucoside; Cya-3-O-glu, cyanidin 3-O-glucoside; Cya-3-O-rut, cyanidin 3-O-rutinoside; Del-3-O-glu, delphinidin 3-O-glucoside; Del-3-O-rut, delphinidin 3-O-rutinoside; Peo-3-O-ara, peonidin 3-O-arabinoside; Peo-3-O-glu, peonidin 3-O-glucoside; Cya-3-O-(6-O-malonyl)-glu, cyanidin 3-O-(6-O-malonyl-beta-D-glucoside); Pel-3-O-rut, pelargonidin 3-O-rutinoside.

To further understand the correlation between the transcriptome and metabolome in *P. virginiana* leaf, we carried out correlation analysis of DEGs and DAMs during leaf color change. Many structural genes and metabolites were significantly enriched in phenylpropanoid, flavonoid and anthocyanin biosynthesis, indicating that metabolite accumulation was specifically regulated by the DEGs. For correlation analysis, genes with coefficients of r > 0.8 or < −0.8 were selected. According to KEGG enrichment, 3433 DEGs are associated with 30 metabolites, in which 2090 (60.9%), 1388 (40.4%), 1324 (38.6%) and 1014 (29.5%) of the DEGs were significantly associated with malvidin 3-O-glucoside, pelargonidin 3-O-glucoside, malvidin 3,5–diglucoside and cyanidin 3-O-arabinoside, respectively (Table S10). In total, 1108 DEGs were in common correlation with malvidin 3-O-glucoside and pelargonidin 3-O-glucoside, indicating that these two metabolites have similar accumulation pattern (Figure S6). The correlation network was used to the find the regulatory correlation between metabolites and genes involved in phenylpropanoid and flavonoid biosynthetic pathways in GL vs. PRL (Figure 8), demonstrating the strong correlations between the DEGs and metabolites. MYB TFs and some key structural genes (such as *CHS* and *CHI*) were significantly positively correlated with some metabolites, especially malvidin 3-O-glucoside and pelargonidin 3-O-glucoside, indicating that these TFs and genes play a key role in phenylpropanoid and flavonoid biosynthesis.

### 2.10. RT-qPCR Validation of DEGs in Transcriptome Data

**Figure 9:**
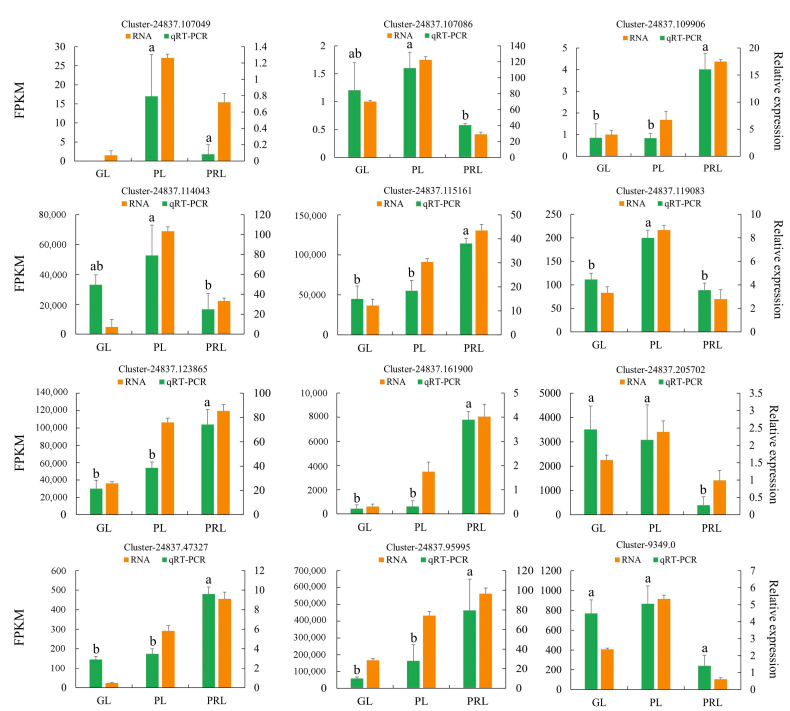
Quantitative real-time PCR verification of expression levels of 12 DEGs identified by RNA sequencing. The *y*-axis on the left represents the FPKM value obtained by RNA-seq. The *y*-axis on the right shows the relative gene expression levels (2^−ΔΔ*C*t^) analyzed by qRT-PCR. The *x*-axis represents the different leaves samples. Bars with different lowercase letters are significantly different (*p* < 0.05). GL, green leaves; PL, purple leaves; PRL, purple-red leaves. FPKM represents the fragments per kilobase per million.

In order to validate the transcriptome data, 12 DEGs (eight upregulated and four downregulated) related to phenylpropanoid, flavonoid and anthocyanidin biosynthesis were selected for expression analysis in GL, PL and PRL by using qRT-PCR. The relative expression of these candidate genes was similar to the RNA-seq results, indicating consistency in the RNA-seq data obtained by Illumina sequence and qRT-PCR (Figure 9).

## 3. Discussion

*P. virginiana* is a colorful tree with great ornamental value because of its attractive shape, colorful leaves and resistance to biotic and abiotic stress. Its distinctive leaf color change (from green to purple and then to purplish-red) is regulated by specific gene expression and variation in metabolite abundance. Previous studies focused on photosynthesis, cultivation techniques and leaf pigment content [4,5]. Analysis of the regulatory mechanisms and abundant metabolites using multi-omics methods to study leaf color change has been lacking. A combined analysis of metabolome and transcriptome data was carried out to elucidate the mechanisms that determine metabolite accumulation during leaf color change and to provide mechanism-based information for genetic improvement of *P. virginiana*.

### 3.1. Pigments Accumulation in Different Color Leaves

The formation of leaf color is complex and based on accumulation, content, biosynthesis, distribution and types of intracellular pigments, the anthocyanins, chlorophylls and carotenoids. Under natural conditions, the leaves at the top of the branches of *P. virginiana* are green. Then, the leaves gradually change from green to light purple in early June and finally to purplish-red. The intracellular pigments showed significant differences in all color stages, suggesting that they play a crucial role in leaf color change (Figure S1). Leaf color was closely related to the types and proportions of pigment in purple-leaf plants. The content of flavonoids in purple leaves was higher than in green leaves, while the chlorophyll and carotenoid contents were lower [17], which are results that are confirmed in the present study. In general, the content of anthocyanin had a significant negative relation with chlorophyll in colorful tree in colorful tree species [18]. In the present study, our results showed that chlorophyll was the crucial factor that affected GL, while the dominant pigments in PRL was anthocyanin. Particularly, the expression level of a vast majority of structural genes related to chlorophyll biosynthesis in GL were higher than that in PRL, while the high accumulation of anthocyanin corresponded to the structural gene involved in anthocyanin biosynthesis in PRL. This result was consistent with previous evidence suggesting that chlorophyll plays a key role in photosynthesis, while anthocyanin prevents damage to the photosynthetic system [19]. Furthermore, the leaves were collected in June, and the light intensity and temperature were significantly higher than that in May, which may result in leaf oxidative damage and further influences the chlorophyll contents between GL/PL and PRL. This might explain why the content of anthocyanin had a significant negative relation with chlorophyll. Anthocyanins are one of the most important plant pigments that determine the color of plant organs. While 30 anthocyanidins were detected and quantified during leaf color change, 11 metabolites, including cyanidin-3-O-glu, cyanidin-3-O-rut, delhpinidin-3-O-rut, delphinidin-3-O-(6-O-malonyl)-glu, cyanidin-3-O-(6-O-malonyl)-glu, malvidin-3-O-glu, pelargonidin-3-O-glu, peonidin-3-O-glu, petunidin-3-O-glu, cyanidin-3,5-O-diglu and pelargonidin-3-O-(6-O-malonyl) -glu, were identified as significantly upregulated in the purple-red leaf (PRL) of *P. virginiana* but not in the green leaf (GL), which enables the leaf to turn purple. These compounds have been reported in coloration studies of leaves or fruits of many plants [6,20]. Similarly, in the correlation network, the majority of structural genes involved in anthocyanin biosynthesis were correlated with malvidin and pelargonidin. In particular, malvidin 3-O-glucoside and pelargonidin 3-O-glucoside were the predominant anthocyanins associated with numerous DEGs (*r* > 0.8 or < −0.8) and were considered to be the anthocyanins related to purple-red pigmentation in *P. virginiana* (Table S6). Similar results were reported in tea plants, passion fruit and elephant grass, with purple leaves or fruit [21,22,23,24].

### 3.2. The Genes Involved in Anthocyanin Biosynthesis in Leaves of P. virginiana

Leaf color is a significant factor in ornamental value [25]. Leaf color is related to some key structural genes in flavonoid and anthocyanin biosynthesis such as *PAL*, *CHS*, *CHI*, *F3H*, *DFR*, *ANS* and *UFGT* and regulatory factors such as MYB, bHLH, WD40 and bZIP [26,27]. Anthocyanins are abundant secondary metabolites produced from phenylalanine through a series of enzymatic reactions, which are further modified by acylation, methylation and glycosylation until they are finally stored in vacuoles [28]. Anthocyanins are found in six groups including pelargonidin, cyanidin, delphinidin, peonidin, petunidin and malvidin, in which the top three pigments are most widespread in plant tissues [29]. The anthocyanins of purple-red leaves compared with green leaves are rich in cyanidin 3-O-arabinoside, cyanidin 3-O-galactoside, cyanidin 3-O-glucoside, cyanidin 3-O-rutinoside and pelargonidin 3-O-glucoside. In anthocyanidin biosynthesis in *P. virginiana*, 11 key structural genes were activated. Among them, the expressions of DEGs encoding *CHS*, *CHI*, *F3H*, *DFR* and *ANS* were higher in PRL than in GL, which may result in higher anthocyanidin during the purple-red leaf stage. *ANS* genes play essential roles in anthocyanin biosynthesis, because they directly convert leucoanthocyanidins into colored anthocyanidins [30]. The lack of *ANS* and *DFR* activity results in the loss of pigmentation [14]. Two *ANS* genes (*Cluster-24837.114104* and *Cluster-24837.114106*) and one *DFR* genes (*Cluster-24837.115161*) were significantly upregulated in PRL, which might result in the accumulation of colored anthocyanins in purple-red leaves. The *UFGT* gene can convert anthocyanins to a more stable water-soluble state as the last step of flavonoid pathway [31]. With high expression of two *UFGT* genes (*Cluster-24837.111509* and *Cluster-24837.123865*), the leaf color gradually turns purple-red, which is consistent with previous studies. These genes are candidates for the formation of purple-red leaves in *P. virginiana*. We also identified three types of genes for anthocyanin biosynthesis including *BZ1*, *3GGT* and *3AT*, which may be involved in acylation, glycosylation, transport and deposition of anthocyanidins [23,32]. *BZ1* (anthocyanidin 3-O-glucosyltransferase) was highly expressed in purple-red leaves and their downstream metabolites (Figure 6A). Pelargonidin 3-O-glucoside, cyanidin 3-O-glucoside and delphinidin 3-O-glucoside were also upregulated in PRL (Figure 6B). *BZ1* could be a regulator of anthocyanidin abundance during leaf color change. However, the expression of the *3GGT* and *3AT* gene decreased and showed the same variation as leaf color change. Their downstream products were not found in purple-red leaves, suggesting that they may be silenced. The results are consistent with the leaf color phenotypes and the corresponding metabolites.

### 3.3. The Genes Involved in Chlorophyll Biosynthesis in Leaves of P. virginiana

Chlorophyll metabolism is of great significance to plant leaf color change. The accumulation of chlorophyll results in green leaves, while a small amount of chlorophyll will be etiolated. We identified some structural genes that may regulate chlorophyll biosynthesis and accumulation (Figure 7). These genes display high-level expression in green leaves, followed by purple and purple-red leaves, consistent with the chlorophyll content data. Similar results were also found in a study of the gene regulation of chlorophyll abundance in red maple leaves [33]. These genes might participate in chlorophyll biosynthesis. The expression of two *CLH* genes (*Cluster-24837.1383* and *Cluster-24837.8715*) and one *chl-G* gene (*Cluster-24837.111895*) in PRL was higher than that in GL and PL, suggesting that they may negatively regulate chlorophyll biosynthesis in purple-red leaves in *P. virginiana*.

### 3.4. Transcription Factors Related to Anthocyanin Biosynthesis

In addition to its key structural genes, anthocyanin biosynthesis is regulated by several transcription factors (TFs) such as MYB, MADS-box, bHLH, WD40, bZIP and WRKY [34,35]. R2R3-MYB is a core transcription factor of anthocyanin biosynthesis in plants. It activates *PAL*, the initial gene of phenylpropanoid biosynthesis, and consequently increases the abundance of anthocyanins [36]. Particularly, R2R3-MYB interacts with bHLH and WD40 to form the MYB-bHLH-WD40 regulatory complex (MBW) in order to further induce anthocyanin biosynthesis and accumulation in plants [37,38]. MYB and the heterogenous bHLH TFs activate key structural genes for anthocyanin biosynthesis in blueberry [39]. There are protein complexes that negatively regulate key genes in the flavonoid pathway in plants [40,41]. In this study, 18 and 17 MYB genes were upregulated in GL vs. PRL and PL vs. PRL, and they may positively regulate the expression of the majority of the structural genes involved in flavonoid biosynthesis with the MBW complex in the purple-red leaf stage. One *bHLH* gene (*Cluster-24837.158504*) was upregulated and may be a key regulator for anthocyanin accumulation in purple-red leaves. We propose that the purple-red leaf stage in *P. virginiana* might be attributed to the coordinated regulation of *MYB* genes and *bHLH* genes, similar to that recently reported in Artemisia annua L. [42]. Some TFs such as AP2/ERF, C2H2, HD-Zip, WRKY and MADS-box TFs display differential expression during leaf color change, suggesting that they may synergistically regulate the expression of genes in flavonoid biosynthesis, as mentioned in a recent report [43] in which the MYB and HD-Zip TFs have similar expression and may be the key regulators in flavonoid biosynthesis. Similar results were also found in a study of color change in Jujube peel [26].

## 4. Materials and Methods

### 4.1. Materials and Sampling

Plants were cultivated on the campus of Northeast Forestry University (NEFU) (45°43′ N, 126°38′ E), Harbin, Heilongjiang province in China. Fresh leaves with normal growth under good lighting condition outside and clear of pests and pathogens were selected at the same stage of development. In June 2019, green leaves (GL), purple leaves (PL) and purplish-red leaves (PRL) were collected from three different *P. virginiana* individuals and were quickly transported to the laboratory (Figure 1A,B). They were then frozen in liquid nitrogen and stored at −80 °C for further utilization. Each color leaf samples contains three biological repeats. For each repeat, 15 leaves were considered as one mixed sample, and the total weight was at least 3 g. In addition, three biological repeats were also performed for each color leaf in order to measure the physiological index, chlorophyll and carotenoid content.

### 4.2. Measurement of Chlorophyll and Carotenoid Contents

The extractions of chlorophyll and carotenoids were performed as described by Chen et al. [44]. The *P. virginiana* leaves (~0.2 g) for each sample were cut into squares with a side length of 0.2 cm and placed into 50 mL centrifuge tubes. Then, 45 mL of acetone: absolute alcohol (1:1) was added. The centrifuge tubes were placed in the dark for 24 h and shaken four times before colorimetric determination. The absorbance of the solution was measured at 646 nm, 663 nm and 470 nm. Chlorophyll a (Chl-a), chlorophyll b (Chl-b) and carotenoid content (Car) were calculated by using the following formulae: Chl-a = (12.21 × A663 − 2.81 × A646) × V/1000 × W; Chl-b = (20.13 × A646 − 5.03 × A663) × V/1000 × W; and Car = (4.4 × A470 − 0.01 × Chla-0.45 × Chlb) × V/1000 × W, where A represents absorbance, V represents the volume (ml) and W represents the leaf fresh weight (g). Three technical replications were measured.

### 4.3. RNA-Seq and Function Annotation

RNA isolation, cDNA library construction and sequencing were performed following the methods described previously [45]. The amount of 0.1 g of leaf tissue was used for RNA extraction. Quantification, purity and integrity of total RNA were estimated using the Qubit 2.0 Fluorimeter (Life Technologies, Carlsbad, CA, USA), NanoPhotometer^®^ spectrophotometer (IMPLEN, Westlake Village, CA, USA) and an Agilent 2100 bioanalyzer (Agilent Technologies, Santa Clara, CA, USA). PolyA-mRNA was isolated from total RNA by using oligo-dT magnetic beads. A fragmentation buffer was added to break the RNA into short segments, and the first strand cDNA was synthesized by reverse transcriptase. From the PCR amplification of the cDNA, nine libraries from three groups were constructed for further RNA-seq. All of the libraries were sequenced on an Illumina HiSeq platform, which generated the raw data of 150 bp long paired-end reads.

The raw reads were filtered with the fastp software (version 0.12) to obtain high-quality clean sequences. De novo assembly was performed using Trinity software. Gene functional annotation of the assembled unigenes was performed based on the public databases, including Nr (NCBI non-redundant protein sequences), Pfam (Protein family), COG (Clusters of Orthologous Groups of proteins), KOG (euKaryotic Ortholog Groups), Swiss-Prot (A manually annotated and curated protein sequence database), GO (Gene Ontology), KEGG (Kyoto Encyclopedia of Genes and Genomes) and Trembl. Fragments per kilobase of transcript per million fragments mapped (FPKM) was used to calculate the expression level of each gene by using RSEM software.

### 4.4. Analysis of Differentially Expressed Genes (DEGs), GO and KEGG Enrichment

For differential expression, the count matrix of different comparison groups was calculated by using the DESeq2 R package (1.20.0). The resulting *p* values were adjusted using the Benjamini and Hochberg’s false discovery rate (FDR). Genes with an adjusted *p*-value < 0.05 and |log2 Fold Change (FC)| ≥ 1 were considered differentially expressed genes (DEGs). Gene Ontology (GO) analysis of the DEGs was implemented by the GOseq R package based on the Wallenius non-central hyper-geometric distribution. The KOBAS 2.0 software was used to test the statistical enrichment of (DEGs) in KEGG pathways.

### 4.5. Sampling Preparation and Metabolite Extraction

The leaf samples for metabolite extraction were quickly freeze-dried and crushed using a mixer mill (MM 400, Retsch) with zirconia beads for 1.5 min at 30 Hz. The amount of 50 mg of powder was extracted with 0.50 mL methanol/water/hydrochloric acid (799:200:1, V/V/V). The extracts were vortexed for 10 min, followed by sonication for 10 min. The solution was centrifuged at 12,000× *g* for 3 min at 4 °C to collect the supernatants, which were filtered (PTFE, 0.22 μm) (ANPEL, Shanghai, China) before LC-MS/MS analysis. LC-MS/MS analysis was performed by Wuhan MetWare Biotechnology Co., Ltd., (www.mettware.cn) (Accessed 30 November 2020) using the methods of Zou et al. [46].

### 4.6. Metabolite Profiling

Normalized metabolite data from nine leaf samples of three colors were used to compare the metabolites from different leaf colors. Hierarchical Cluster Analysis (HCA) and orthogonal partial least-squares discriminant analysis (OPLS-DA) were processed by the R-package in order to identify the differences between the nine leaf samples. The OPLS-DA model was used to identify the differences of metabolite composition between the samples by variable importance in projection (VIP) values (VIP ≥ 1) and fold change ≥ 2 or ≤ 0.5.

### 4.7. Integrative Analysis of Metabolomics and Transcriptomic Data

Pearson correlations were calculated for the integrative analysis between the various metabolites and the DEGs in anthocyanin biosynthesis. Correlations with a coefficient (*r*) value > 0.8 or < −0.8 were considered to be crucial relationships between the metabolome and transcriptome. To visualize the specific relationships, correlation network analysis was performed using OmicStudio (Hangzhou, China) tools (https://www.omicstudio.cn/tool) (Accessed 5 April 2021).

### 4.8. Quantitative Real-Time PCR (qRT-PCR) Validation

To validate the transcriptome data, the relative expressions of 12 DEGs identified in transcriptome analysis were evaluated by qRT-PCR using three biological and three technical replicates in the analysis. RNA extraction and cDNA synthesis were conducted using the Tiangen total RNA extraction kit (Tiangen, Beijing, China) and PrimeScript RT reagent Kit with gDNA Eraser (TaKaRa, Kyoto, Japan), respectively. The primers for qRT-PCR were designed using the Primer Premier 5 software (http://www.premierbiosoft.com/primerdesign/index.html) (Accessed 20 April 2021). qRT-PCR tests were carried out with the TaKaRa SYBR Green Mix kit (TaKaRa, Beijing, China) using the ABI 7500 Fast Real-Time Detection System. PCR amplification was performed in 20 µL containing 10 µL 2xSYBR Primix ExTaq, 0.8 µL upstream primer, 0.8 µL downstream primer, 0.4 µL Rox-Reference dye, 2 µL cDNA template and 6 µL ddH_2_O under a standard PCR program of 95 °C for 30 s; 40 cycles at 95 °C for 5 s; 60 °C for 35 s; 95 °C for 15 s; and 60 °C for 1 min, followed by 95 °C for 15 s. The relative expression analysis of quantitative data was performed by using 2^−ΔΔ*C*t^ with 18S-rRNA as the reference gene.

## 5. Conclusions

In this study, the molecular regulatory mechanisms of leaf color change in *P. virginiana* were characterized by combining metabolome and transcriptome data. The biosynthesis and accumulation of pigments in the formation of leaf color depend on the structural genes for anthocyanin and anthocyanidin metabolite biosynthesis, especially malvidin 3-O-glucoside and pelargonidin 3-O-glucoside. The biosynthesis of chlorophyll was apparent and the temporal expression of key genes was identified. The expression of flavonoid biosynthesis genes (PAL, CHS and CHI) and their transcriptional regulators (MYB, HD-Zip and bHLH) increased during the purple-red periods. In addition, the expression of genes encoding enzymes in the anthocyanin biosynthetic pathway, UDP glucose-flavonoid 3-O-glucosyl-transferase (UFGT) and anthocyanidin 3-O-glucosyltransferase (BZ1), was substantially higher than in leaves of other color and may be related with the purple-red color change. These results provide novel insights into the molecular regulation of anthocyanidin biosynthesis and accumulation in the process of leaf color change in *P. virginiana*. Thus, these results may facilitate genetic modification or selection for further improvement in ornamental qualities of *P. virginiana*.

## Data Availability

Transcriptome sequencing data are available in the SRA database of the National Center for Biotechnology Information (NCBI) under the accession number PRJNA716414.

## Supplementary Materials

The following are available online at https://www.mdpi.com/article/10.3390/ijms221910697/s1, Figure S1: Chlorophyll a (a), Chlorophyll b (b), Chlorophyll a + b (c) and carotenoid content (d) of different color leaves of *P. virginiana*. Error bars show standard error (SE) of the mean. GL, green leaves; PL, purple leaves; PRL, purple-red leaves. Different lowercase letters above the error bars indicate significant difference of correlation at 0.05 level (one-way ANOVA, *p*-value < 0.05); Figure S2: The HPLC ions chromatogram. The *x*-axis represents the retention time of detection. The *y*-axis represents the ions intensity; Figure S3: Cluster analysis of identified metabolites in the present study. Cluster analyses were performed by the k-means methods. The marker on the right side of heatmap represents the names of each anthocyanin composition obtained by high-performance liquid chromatography (HPLC). Color scale from green to red in the heatmap represents the normalized metabolite contents using Row Z-score. GL, green leaves; PL, purple leaves; PRL, purple-red leaves; Figure S4: Principal component analysis based on FPKM data. GL, green leaves; PL, purple leaves; PRL, purple-red leaves. The *x*-axis represents the PC1, and the *y*-axis represents the PC2; Figure S5: Differential expressed genes (DEGs) during leaf color change. (A) Volcano plots of DEGs between GL vs. PL. (B) Volcano plots of DEGs between GL vs. PRL. (C) Volcano plots of DEGs between PL vs. PRL. (D) Venn diagram of all shared DEGs between three compare groups (GL vs. PL, GL vs. PRL and PL vs. PRL). Red, green and blue dots represent upregulated, downregulated and no-regulated genes, respectively. GL, green leaves; PL, purple leaves; PRL, purple-red leaves; Figure S6: Venn diagram of DEGs that are associated with Mal-3-O-glu and Pel-3-O-glu. The correlation of gene with metabolites was identified with coefficients r > 0.8 or < −0.8; Table S1: The number of differentially accumulated metabolites among different groups; Table S2: The results of differentially of accumulated metabolites; Table S3: Statistics of transcriptome data; Table S4: The annotation information of unigenes; Table S5: Differentially expressed genes in leaves of *P. virginiana*; Table S6: KEGG enrichment in different groups; Table S7: The FPKM value of identified MYB, AP2/ERF-ERF and bHLH TFs in anthocyanins biosynthetic pathway. Table S8: Differentially expressed TFs in different groups; Table S9: Statistic of differentially expressed TFs; Table S10: Correlation statistics of DEGs and metabolites.
