# Supplementary material for: Molecular and Metabolic Insights into Anthocyanin Biosynthesis for Leaf Color Change in Chokecherry (Padus virginiana)
Source: Int J Mol Sci. 2021 Oct 2;22(19):10697. doi: 10.3390/ijms221910697 (PMC8509056; doi:10.3390/ijms221910697)
Supplement: Supplementary file 1 [file ijms-22-10697-s001.zip › ijms-1339997 -Supplementary Figures.pdf]

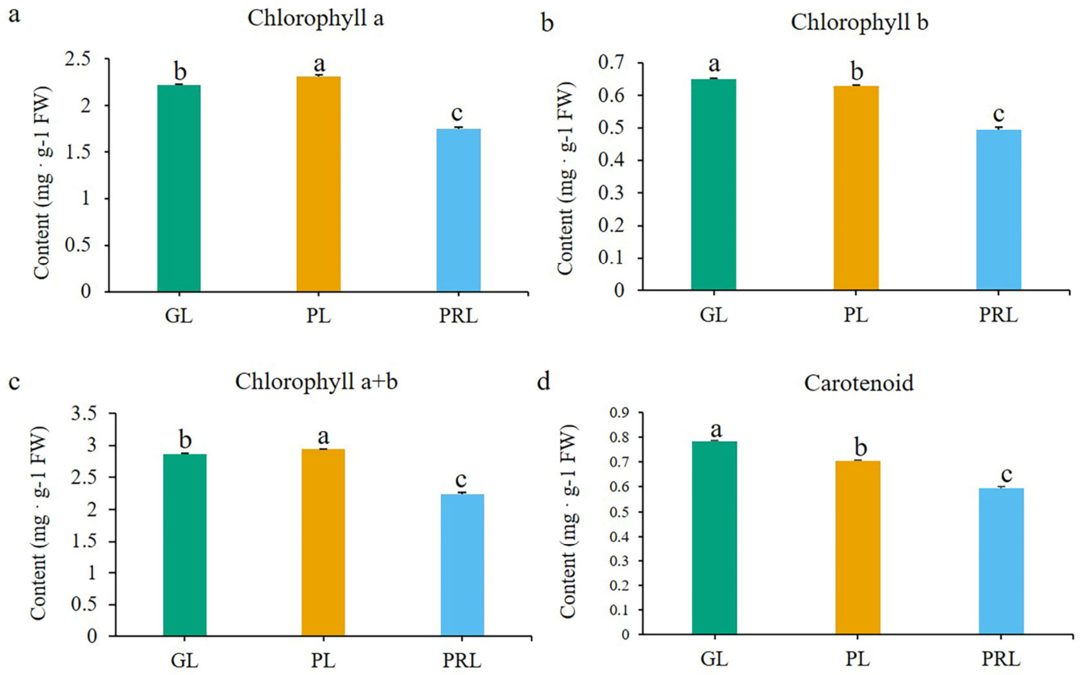

Figure S1. Chlorophyll a (a), Chlorophyll b (b), Chlorophyll a+b (c) and carotenoid content (d) of different color leaves of *P. virginiana*. Error bars show standard error (SE) of the mean. GL, green leaves; PL, purple leaves; PRL, purple-red leaves. Different lowercase letters above the error bars indicate significant difference of correlation at 0.05 level (One way ANOVA, p-value < 0.05).

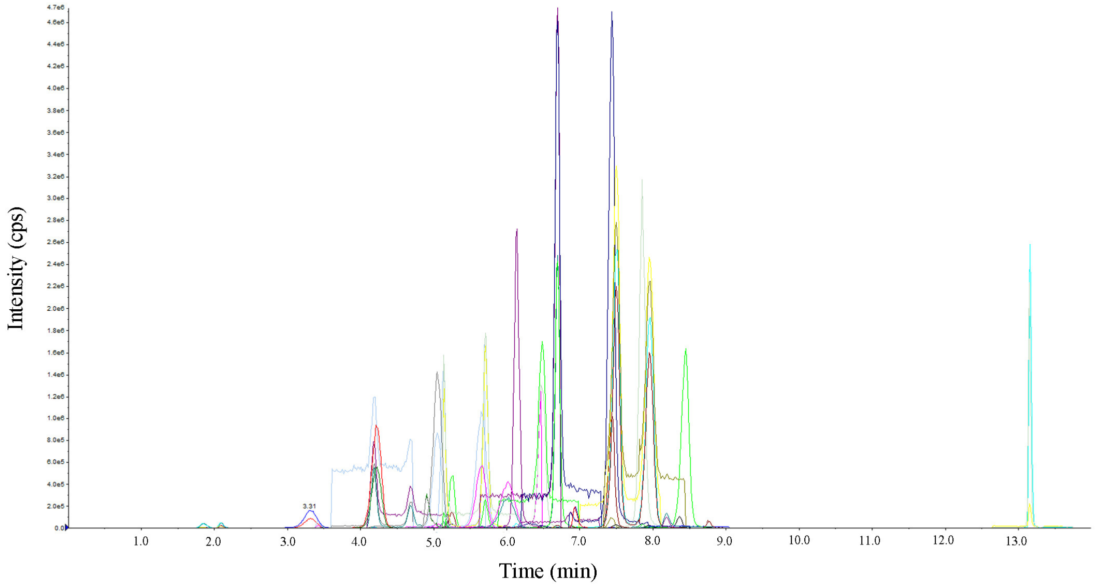

Figure S2. The HPLC ions chromatogram. The x-axis represents the retention time of detection. The y-axis represents the ions intensity.

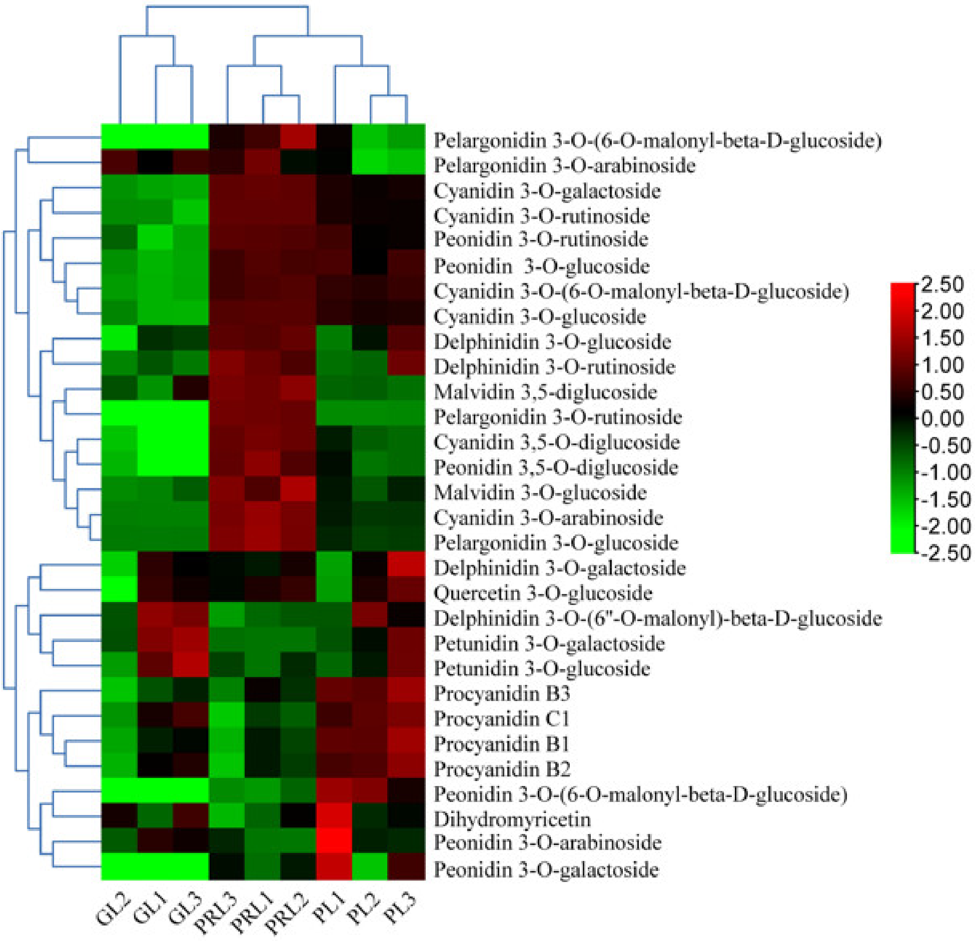

Figure S3. Cluster analysis of identified metabolites in present study. Cluster analyses was performed by the k-means methods. The marker on the right side of heatmap represents the names of each anthocyanin composition obtained by high-performance liquid chromatoraphy (HPLC). Color scale from green to red in the heatmap represents the normalized metabolite contents using Row Z-score. GL, green leaves; PL, purple leaves; PRL, purple-red leaves.

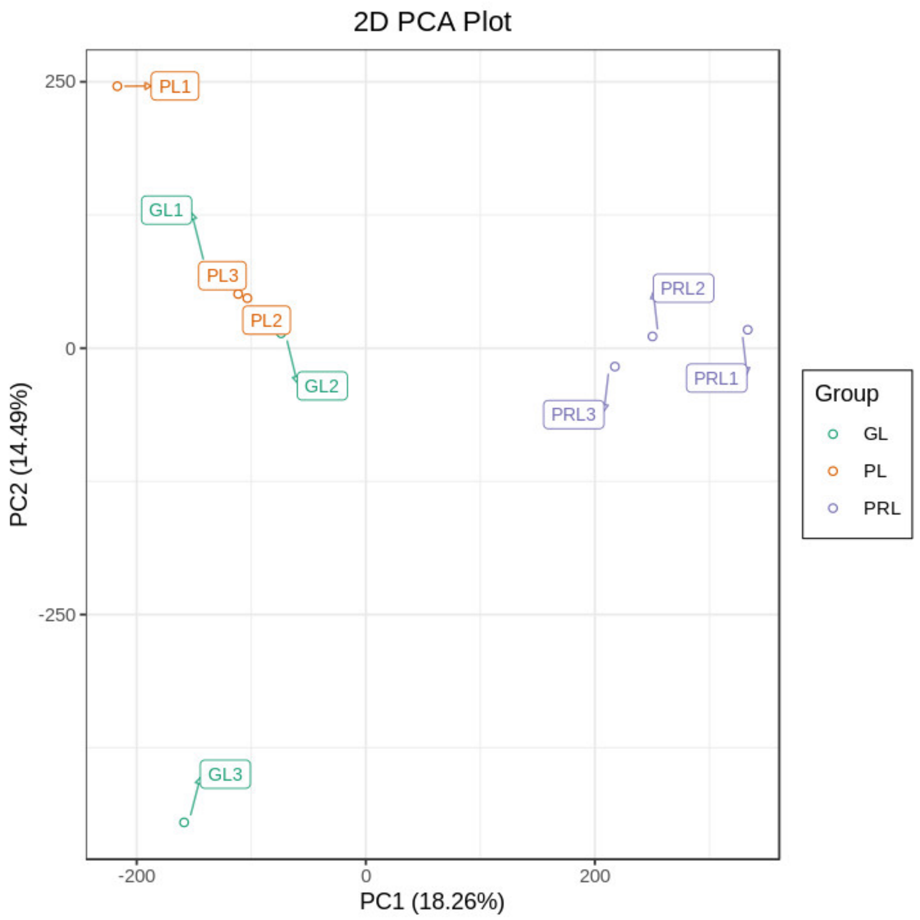

Figure S4. Principal component analysis based on FPKM data. GL, green leaves; PL, purple leaves; PRL, purple-red leaves. The x-axis represents the PC1, and the y-axis represents the PC2.

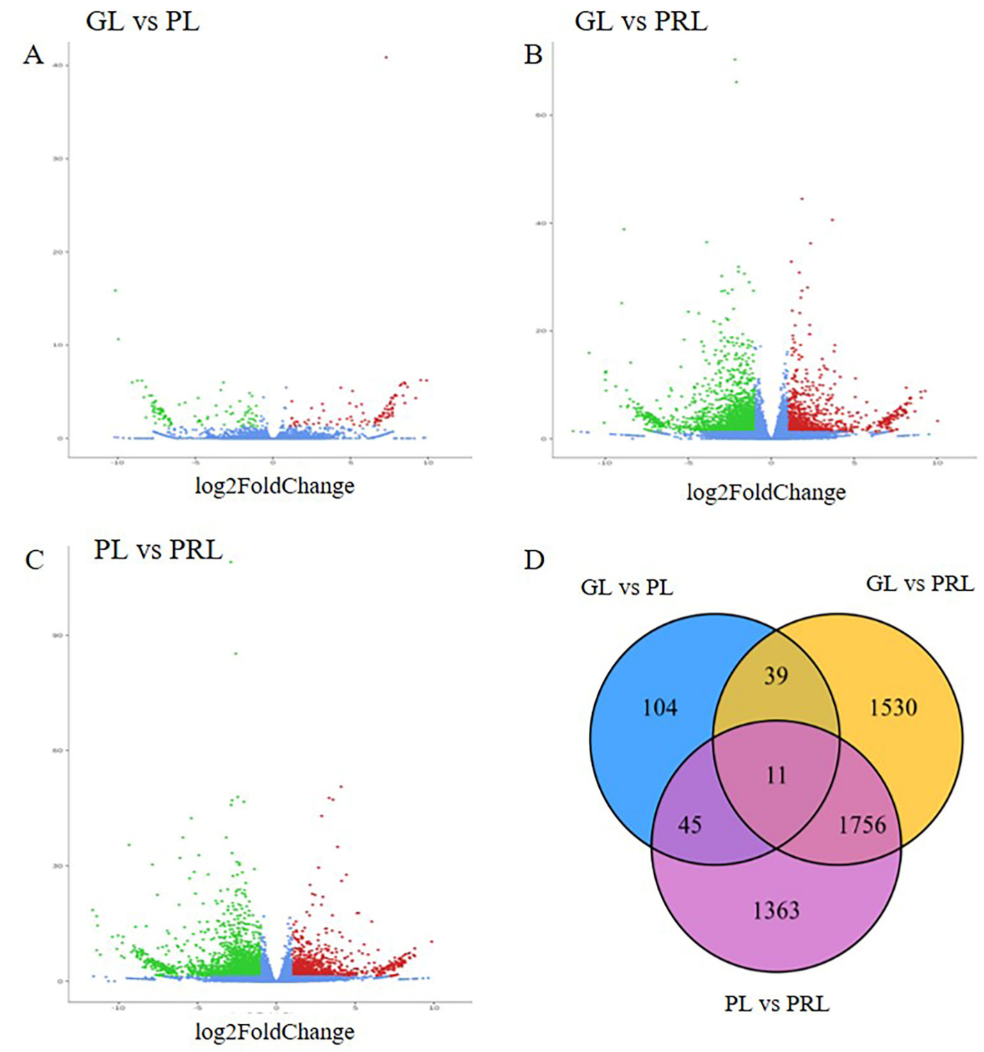

Figure S5. Differential expressed genes (DEGs) during leaf color change. (A) Volcano plots of DEGs between GL vs PL. (B) Volcano plots of DEGs between GL vs PRL. (C) Volcano plots of DEGs between PL vs PRL, (D) Venn diagram of all share DEGs between three compare groups (GL vs PL, GL vs PRL and PL vs PRL). Red, green and blue dots represent up-regulated, down-regulated and no-regulated genes, respectively. GL, green leaves; PL, purple leaves; PRL, purple-red leaves.

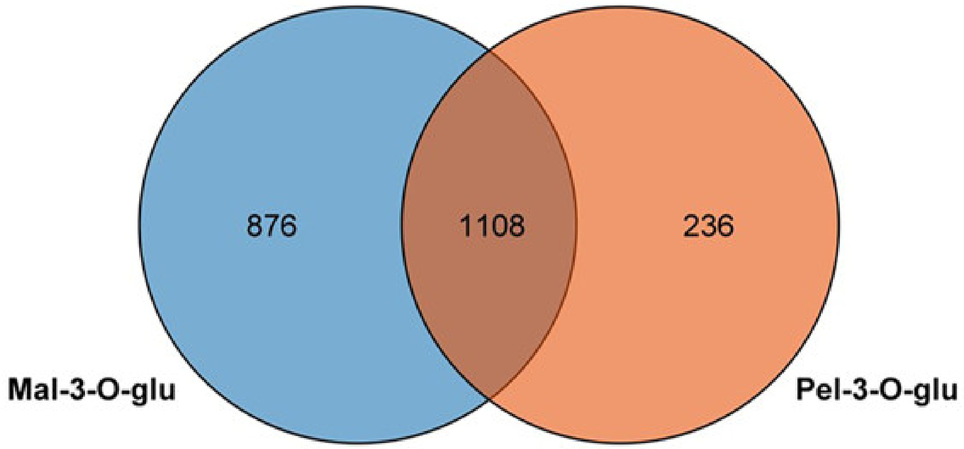

Figure S6. Venn diagram of DEGs that are associated with Mal-3-O-glu and Pel-3-O-glu. The correlation of genes-metabolites was identified with coefficients r > 0.8 or < -0.8.
